# Neuroimaging and serum biomarkers of neurodegeneration and neuroplasticity in Parkinson’s disease patients treated by intermittent theta-burst stimulation over the bilateral primary motor area: a randomized, double-blind, sham-controlled, crossover trial study

**DOI:** 10.3389/fnagi.2023.1258315

**Published:** 2023-10-05

**Authors:** Raúl Rashid-López, Paloma Macías-García, F. Luis Sánchez-Fernández, Fátima Cano-Cano, Esteban Sarrias-Arrabal, Florencia Sanmartino, Constantino Méndez-Bértolo, Elena Lozano-Soto, Remedios Gutiérrez-Cortés, Álvaro González-Moraleda, Lucía Forero, Fernando López-Sosa, Amaya Zuazo, Rocío Gómez-Molinero, Jaime Gómez-Ramírez, José Paz-Expósito, Guillermo Rubio-Esteban, Raúl Espinosa-Rosso, Álvaro J. Cruz-Gómez, Javier J. González-Rosa

**Affiliations:** ^1^Psychophysiology and Neuroimaging Group, Institute of Biomedical Research Cadiz (INiBICA), Cadiz, Spain; ^2^Department of Neurology, Puerta del Mar University Hospital, Cadiz, Spain; ^3^Department of Psychology, University of Cadiz, Cádiz, Spain; ^4^Department of Radiodiagnostic and Medical Imaging, Puerta del Mar University Hospital, Cadiz, Spain; ^5^Department of Neurology, Jerez de la Frontera University Hospital, Jerez de la Frontera, Spain

**Keywords:** Parkinson’s disease, motor and nonmotor symptoms, transcranial magnetic stimulation, intermittent theta-burst stimulation, structural magnetic resonance imaging, functional connectivity, serum biomarkers, neuroplasticity

## Abstract

**Background and objectives:**

Intermittent theta-burst stimulation (iTBS) is a patterned form of excitatory transcranial magnetic stimulation that has yielded encouraging results as an adjunctive therapeutic option to alleviate the emergence of clinical deficits in Parkinson’s disease (PD) patients. Although it has been demonstrated that iTBS influences dopamine-dependent corticostriatal plasticity, little research has examined the neurobiological mechanisms underlying iTBS-induced clinical enhancement. Here, our primary goal is to verify whether iTBS bilaterally delivered over the primary motor cortex (M1) is effective as an add-on treatment at reducing scores for both motor functional impairment and nonmotor symptoms in PD. We hypothesize that these clinical improvements following bilateral M1-iTBS could be driven by endogenous dopamine release, which may rebalance cortical excitability and restore compensatory striatal volume changes, resulting in increased striato-cortico-cerebellar functional connectivity and positively impacting neuroglia and neuroplasticity.

**Methods:**

A total of 24 PD patients will be assessed in a randomized, double-blind, sham-controlled crossover study involving the application of iTBS over the bilateral M1 (M1 iTBS). Patients on medication will be randomly assigned to receive real iTBS or control (sham) stimulation and will undergo 5 consecutive sessions (5 days) of iTBS over the bilateral M1 separated by a 3-month washout period. Motor evaluation will be performed at different follow-up visits along with a comprehensive neurocognitive assessment; evaluation of M1 excitability; combined structural magnetic resonance imaging (MRI), resting-state electroencephalography and functional MRI; and serum biomarker quantification of neuroaxonal damage, astrocytic reactivity, and neural plasticity prior to and after iTBS.

**Discussion:**

The findings of this study will help to clarify the efficiency of M1 iTBS for the treatment of PD and further provide specific neurobiological insights into improvements in motor and nonmotor symptoms in these patients. This novel project aims to yield more detailed structural and functional brain evaluations than previous studies while using a noninvasive approach, with the potential to identify prognostic neuroprotective biomarkers and elucidate the structural and functional mechanisms of M1 iTBS-induced plasticity in the cortico-basal ganglia circuitry. Our approach may significantly optimize neuromodulation paradigms to ensure state-of-the-art and scalable rehabilitative treatment to alleviate motor and nonmotor symptoms of PD.

## Introduction

Parkinson’s disease (PD) is increasing more rapidly than that of other neurological conditions, with the disease affecting mostly middle-aged persons (Dorsey et al., 2018). Aside from the inherent complexity of the pathophysiology itself, the annually increasing prevalence of PD poses a further challenge for public health systems in aging societies (Rodríguez-Blázquez et al., 2015), which must offer treatments that mitigate the medical, social, and personal impacts of this disease as well as identify clinical markers of treatment effectiveness. Clinically, PD is characterized by the presence of heterogeneous symptoms, predominantly consisting of typical motor symptoms, such as bradykinesia, tremor, rigidity, gait disorder, and postural instability, which have a significant clinical impact on patients and relatives (Weiner, 2006; Moustafa et al., 2016). Nonmotor features, such as cognitive impairment, sleep disorders, and mood and affect changes, also occur frequently, with a considerable impact on disability and health-related quality of life (Chaudhuri et al., 2006; Schapira et al., 2017; Macías-García et al., 2022). Pathologically, the loss of dopaminergic neurons and decreased striatal dopamine levels have been proposed as the mechanisms behind motor deficits in PD (Rosin et al., 2007). This denervation induces significant changes in synaptic functioning, increases maladaptive neural activity along cortico-striatal-pallido-thalamic-cortical circuits (Kish et al., 1988; Salenius et al., 2002; Herz et al., 2014; Weiss et al., 2015), and alters glucose metabolism and blood flow in the brain (Obeso et al., 2008; Nandhagopal et al., 2011; Stoessl et al., 2014). The pathology also features nonspecific alterations in gray matter (GM) and white matter (WM) at structural and functional levels in the BG-thalamo-cortical circuit (Lewis et al., 2016; Sterling et al., 2016; Filippi et al., 2020), resulting in progressive atrophy in various brain regions from onset to the intermediate phases (Filippi et al., 2020; Sarasso et al., 2021).

Dopamine-based treatments such as levodopa are commonly used to alleviate motor symptoms PD; the principal drawbacks of these replacement treatments are the limited time window for their use and the fact that they can lead to dyskinesias, motor fluctuations, and cognitive problems (Lang and Espay, 2018). Alternative therapies, such as noninvasive brain stimulation and repetitive transcranial magnetic stimulation (rTMS), are being explored for the treatment of motor and nonmotor symptoms in PD patients. Current evidence suggests that while low-frequency rTMS (≤1 Hz) has an inhibitory impact (Chen et al., 1997), high-frequency rTMS (≥ 5 Hz) induces excitability in the cortex (Pascual-Leone et al., 1994; Kobayashi and Pascual-Leone, 2003). Recently, theta-burst stimulation (TBS), an rTMS pattern involving bursts of high-frequency stimulation that mimics neural oscillatory patterns, has been shown to increase or decrease cortical excitability depending on the presence of an intertrain interval, i.e., whether the protocol is continuous (cTBS) or intermittent (iTBS; Di Lazzaro et al., 2005; Huang et al., 2005; Gamboa et al., 2011). The evaluation of this technique as a potential therapeutic modality with promising outcomes in neurorehabilitation in a variety of neurological and neuropsychiatric disorders has been prompted by the finding that the modulatory effects of both rTMS and TBS induce neuroplasticity that lasts beyond the stimulation period (Huang et al., 2017; Kricheldorff et al., 2022). Current evidence suggests that the application of iTBS and cTBS patterns to the motor cortex can facilitate or depress corticospinal excitability, respectively, and induce synaptic plasticity, as revealed in experimental models of PD (Chen and Udupa, 2009; Ghiglieri et al., 2012; Natale et al., 2021) and multiple studies on cortical plasticity in humans, in which the effect lasted from minutes to hours after administration (Di Lazzaro et al., 2005; Huang et al., 2005, 2017; Wischnewski and Schutter, 2015). Thus, the majority of PD studies suggest that cTBS over the supplementary motor area (SMA) has strong potential to enhance motor function and that iTBS over the motor primary area (M1) or the dorsolateral prefrontal cortex may improve specific aspects of motor performance, gait, and nonmotor symptoms in PD (Cheng et al., 2022). Nevertheless, despite the undeniable advantages of TBS over conventional TMS, the conclusions of these studies have some inconsistencies, particularly in terms of the duration and variability of the clinical effects in this patient population, the target of stimulation, and the role of dopaminergic medication use.

Yet, the potential advantages that iTBS brings to PD patients remain an open question, particularly regarding the degree to which iTBS-induced excitability and plasticity chances are confined to the cortical level, influencing GABAergic and fast-spiking interneurons and modulating inhibitory control of pyramidal cell output activity (Di Lazzaro et al., 2005; Benali et al., 2011), or whether these changes may be accompanied by changes at subcortical and striatal locations (Ghiglieri et al., 2012). On this topic, preclinical and human experiments have shown that iTBS enhances spontaneous neuronal firing and induces long-term potentiation (LTP)-like changes in M1 (Huang et al., 2005; Ziemann and Siebner, 2008; Wischnewski and Schutter, 2015), which might directly or indirectly facilitate the modulation of striatal dopamine release (Strafella et al., 2003, 2005; Ohnishi et al., 2004) and other nondopaminergic pathways (Aceves-Serrano et al., 2022), supporting the idea that a rescue of corticostriatal plasticity and a recovery of corticostriatal long-term depression (LTD), which becomes impaired in PD, may underlie the recovery of motor control in PD patients after M1-iTBS (Cacace et al., 2017; Natale et al., 2021).

At present, more research effort is needed to develop functional and structural biomarkers that determine the efficacy of iTBS-related clinical interventions and to characterize small changes in motor and nonmotor symptoms; many questions remain regarding the pattern of brain structural changes following TBS in PD patients (Huang et al., 2017; Ji et al., 2021). Likewise, more work is needed to characterize the underlying neural mechanisms by which TBS influences macro- and microstructural damage and whether these types of damage represent different neurodegenerative progresses. Regarding these matters, a growing body of research has shown that rTMS and TBS may modulate or induce neurological changes involving inflammation, neuroprotection and neurodegeneration, both during and after treatment (Bashir et al., 2022), but the precise neurobiological mechanism underlying the potential neurorestorative effects of iTBS in patients with PD is still not fully understood (Suppa et al., 2016; Natale et al., 2021). Furthermore, although the direct involvement of cortico-BG pathways in motor symptom alleviation in PD patients after TBS therapy has been suggested (Cárdenas-Morales et al., 2010; Chen et al., 2022), it remains unclear how structural and functional brain changes are ultimately associated with serum biomarkers of neurodegeneration and neuroplasticity and how they contribute to clinical improvement.

To validate the therapeutic effectiveness of iTBS as an adjunct to standard dopaminergic therapy on motor and nonmotor symptoms in PD patients on medication, we designed this randomized, double-blind, sham-controlled crossover study protocol in which iTBS is applied over the bilateral M1. Additionally, given that few studies have explored the neuronal correlates of the clinical and/or neuroprotective effects of iTBS, we will also investigate the potential neurobiological mechanisms of their therapeutic actions by assessing the relationships of dynamic brain changes (assessed by structural magnetic resonance imaging [MRI] and resting-state electroencephalography [EEG] and functional MRI [fMRI] data) with blood markers of neurodegeneration and neuroplasticity as well as possible clinical benefits.

Therefore, a major strength of this study will be its examination of the short-term motor and nonmotor impacts of bilateral M1-iTBS in patients with PD by exploiting the current trends in neuroimaging and serum biomarker testing to develop a multimodal architecture that characterizes brain structural and functional integrity in PD patients and its likely relationship with symptom dimensions.

## Methods

### Description of the protocol study design

We designed a single-center, randomized, double-blind, sham-controlled, crossover protocol study on patients with PD; these patients will be recruited at the Puerta del Mar University Hospital of Cadiz. The aim is to investigate the clinical efficacy of bilateral M1 iTBS (administered in 5 sessions) as well as its association with neuropsychological and cortical excitability changes, brain imaging data, and serum biomarkers.

### Recruitment and sample size

The recruitment period will last approximately one and a half years. During this time, we plan to enroll 24 PD patients who meet the inclusion and exclusion criteria. Recruitment will be entirely managed by neurologists from the Movement Disorders Unit at the Puerta del Mar University Hospital. Every candidate patient will undergo a clinical review by a neurologist specializing in movement disorders, who will assess the suitability of each patient for inclusion in this study.

The inclusion criteria for patients will be as follows: (i) diagnosed with PD according to the United Kingdom Parkinson’s Disease Society Brain Bank diagnostic criteria (UK PDSBB; Gibb and Lees, 1988); (ii) disease duration of at least 5 years to reduce the risk of including levodopa-responsive atypical Parkinsonism patients (Lang et al., 2006; Tolosa et al., 2006; Mestre et al., 2014); (iii) disease symptomatology in the ON medication state at a Hoehn y Yahr (H&Y) scale of II–III; (iv) clinical and therapeutic stability in the last 2 months previous to the recruitment period; and (v) aged 45–75 years.

The exclusion criteria will be as follows: (i) lack of a PD diagnosis that meets the UK PDSBB diagnostic criteria; (ii) presence of a serious systemic disease; (iii) presence of severe or moderate cognitive impairment comparable to dementia as indicated by a Mini-Mental Parkinson (MMP) score of ≤24; (iv) any incapacitating psychiatric or other clinical condition that might affect the correct performance of this protocol, such as any dystonia and/or dyskinesia; (v) receipt of amantadine within the previous 60 days; and (vi) any sign of atypical parkinsonism, neurological comorbidities, or history of cranioencephalic traumatism or epilepsy or any other contraindication to neurostimulation with TMS (e.g., magnetic intracranial implant, cardiac pacemaker).

The few TMS or TBS studies using a crossover design (Eggers et al., 2010; Maruo et al., 2013; Bologna et al., 2015; Kim et al., 2015; Vanbellingen et al., 2016; Yokoe et al., 2018; Hill et al., 2020; Brugger et al., 2021) have demonstrated the favorable efficacy of high-frequency rTMS over the M1 on motor symptoms in PD (compared to sham stimulation). The necessary sample size was calculated based on the primary endpoint, i.e., the motor clinical changes as assessed by the UPDRS-III score. Considering these findings and the statistical parameters needed to detect a treatment difference for a 2 × 2 crossover study at a two-tailed *p* value of 0.05 and a power of 80%, the total sample size was calculated to be at least 21 subjects. With an assumed drop-out rate of approximately 20%, we estimated a final minimum sample size of 24 patients.

### Blinding

The study will have a double-blind design to improve the reliability of results. Random distribution of participants to groups will be supervised by an external investigator, who will use computer-generated numbers to encode the assignment of patients to intervention groups; thus, neither the neurologist nor the evaluator(s) will know which group a patient is in. The study aims to achieve allocation concealment by recording the patients’ randomized group assignments in sealed envelopes, with the researchers and patients having no control over the random allocations, and ensuring that no one involved in the study is aware of the treatment allocations. After the patient signs the informed consent form, an envelope will be opened, and treatment will be allocated in a coded form. Patients will be randomly allocated into two groups, namely, active iTBS and sham stimulation, in a 1:1 ratio. Group allocation will be communicated via email to the person responsible for applying the iTBS treatment. This person will not take part in the data analysis or in the post-iTBS evaluation. All patients will be naive to TMS, and the patients will be kept blind to their assigned treatments throughout the process.

### Procedure and intervention

**Figure 1:**
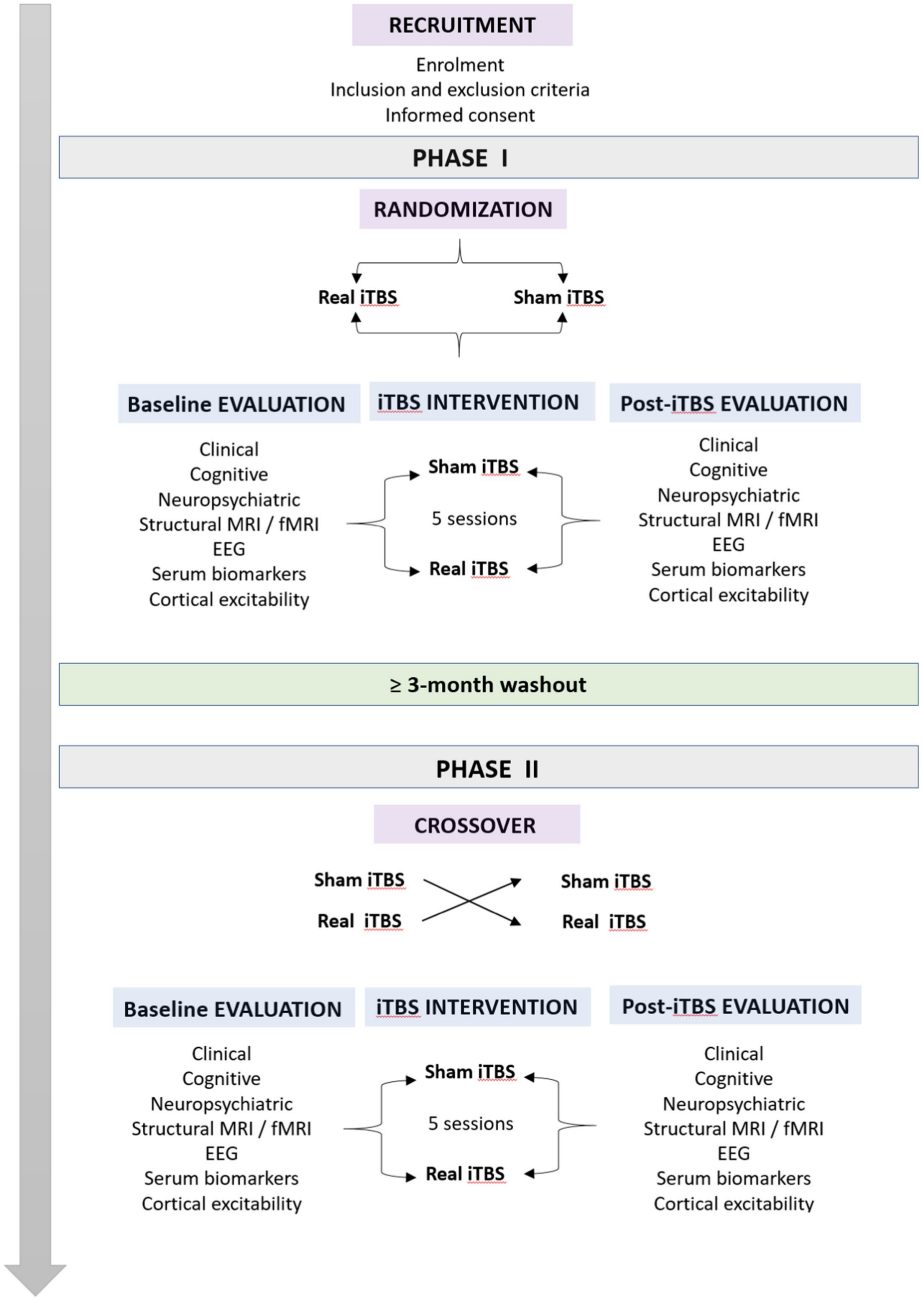
Flowchart of the study design.

Our study will be composed of 2 different phases, Phase I and Phase II, separated by a washout period of at least 3 months. Each phase will consist of 3 stages: baseline evaluation, iTBS intervention, and posttreatment evaluation (Figure 1). The baseline evaluation period will begin once the patients agree to participate in the investigation. From this moment until a maximum of 30 days later, patients will undergo (i) a clinical and motor examination in the ON state, (ii) an extensive cognitive and neuropsychiatric assessment, (iii) a resting-state EEG recording, (iv) structural and functional MRI scans, and (v) blood sample collection. Moreover, immediately following the completion of baseline evaluations and just prior to the start of the iTBS intervention stage, a variety of TMS-EMG cortical excitability measures will be acquired. Once this first stage is fully completed, patients will receive a session of real or sham iTBS over the bilateral M1 once per day for five consecutive days. During the last session of iTBS, TMS-EMG parameters of cortical excitability will be acquired, and patients will undergo a clinical motor evaluation. After the intervention stage, the posttreatment evaluations will be conducted. Subsequently, within a 3–4 day window, resting-state EEG data will be recorded for all participants. A week after the last iTBS session, patients will undergo a second clinical evaluation, and a new blood sample will be collected. Next, on the eighth day posttreatment, structural and functional MRI scans will be acquired. At 2 weeks post-iTBS, a new neuropsychiatric and cognitive assessment will be performed along with a third motor evaluation. Finally, the last visit will occur 28 days after the iTBS intervention, consisting of the fourth and final clinical evaluation (Figure 1). Subsequently, all patients will undergo a washout period of at least 3 months prior to the start of the crossover process and Phase II of the study.

As this study was designed to have a duration of 26 months, the scheduling of all patients will be staggered. According to our schedule, the patient screening period is expected to be completed by month 18. Protocol implementation is expected to be completed by month 30.

#### TMS intervention and neuronavigation

TMS pulses and the iTBS protocol will be delivered using a Magstim Rapid^2^ Magnetic Stimulator (Magstim, Whitland, United Kingdom) connected to a 70 mm air-cooled figure-eight coil (AirFilm® Coil, Magstim, Whitland, United Kingdom). The iTBS treatment will be applied on the motor hand area; real iTBS will have the following parameters: 3-pulse bursts at 50 Hz repeated every 200 ms and delivered as short trains of 10 bursts lasting 2 s and an intertrain silence period of 8 s, for a total of 600 pulses (20 cycles of trains) and a total duration of 190 s. During the five-day iTBS intervention stage, one iTBS session per day will be conducted, with iTBS administered to the bilateral M1 at 80% of the active motor threshold (aMT) in the real condition. Primarily, although the majority of the PD patients are usually bilaterally affected, real or sham iTBS interventions will always start in the M1 contralateral to the most clinically affected hemibody as determined by the MDS-UPDRS-III baseline score. Protocol intensity will be determined on the first day of the intervention and maintained until the last session. The same coil and device will be used for excitability parameter acquisition and iTBS. Sham stimulation during treatment will follow the same procedure except it will be performed with an AirFilm® SHAM coil (Magstim, Whitland, United Kingdom). Both coils are identical in appearance and produce a similar sound and sensation, although the sham coil delivers no stimulation.

Before the iTBS sessions, a full brain and head 3D curvilinear reconstruction will be obtained using a high-resolution 3D T1-weighted anatomical image for neuronavigation (Brainsight, Rogue Research). The T1-weighted image will be rotated and reoriented to the anterior and posterior commissure (AC-PC) plane. Coregistration of the subject to the images will be performed by using four anatomical landmarks: the bridge of the nose, tip of the nose, left preauricular area (LPA) and right preauricular area (RPA). The coil will be placed tangentially to the scalp, and the handle of the coil will be pointing backward and tilted away from the midline of the central sulcus at a 45° angle (Groppa et al., 2012), inducing an anterior–posterior/posterior–anterior current over the bilateral motor hand area, traditionally referred to as the “hand-knob” and corresponding to an inverted omega or epsilon shaped-structure (in the axial plane) located at the middle genu of the central sulcus in the precentral gyrus (Yousry, 1997; Sparing et al., 2008). Because of its large motor cortex representation and low motor unit-to-muscle fiber innervation ratio, the first dorsal interosseous (FDI) muscle of the hand was chosen to quantify TMS-related motor evoked potentials (MEPs; Palmer and Ashby, 1992; Chaves et al., 2021). The optimal position for stimulation will be found by neuronavigated cortical mapping at the time that FDI muscle activation is observed via visual feedback from the electromyography devices and visual inspection. Subsequently, the resting motor threshold (rMT) and aMT, as well as the cortical silent period (cSP) and “20 trials” (20 t), will be determined for the FDI muscle. Again, acquisition of TMS-derived measures of cortical excitability will always be started in the most affected hemisphere (for a more detailed description of the definition and measurement of cortical excitability parameters used in our study, see *Data Collection & Analysis* and *TMS-derived measures of cortical excitability*).

Motor mapping will be performed on a 3 × 3 mm grid above the motor hand area. The center of the grid will be located on the central point of the hand-knob structure such that this area and possible error range are fully covered. For hotspot identification, the central point of the grid will be used for the determination of the minimum suprathreshold intensity that elicits a peak-to-peak MEP > 50 μV for three consecutive trials in the resting state. Subsequently, one TMS pulse will be given at each spot of the grid at 110% of this established intensity. The optimal location or hotspot will be determined as the grid position with the largest amplitude and the shortest latency that provokes movement in the FDI muscle. If these two parameters are not in the same spot, the optimal location will be defined according to the largest amplitude (Charalambous et al., 2019). If no neuroimaging images are available or hotspot identification is not successful after grid mapping, the 10/20 system C1/C2 will be used to locate the motor hand area, which has been demonstrated to offer a more accurate position for the motor hand area than C3/C4 (Silva et al., 2021; Kim et al., 2023). Visually guided and neuronavigated manual mapping will be performed to identify the location of the FDI muscle hotspot. This process will be replicated for both hemispheres.

### Outcome measures

Participants will undergo an extensive assessment of seven areas of interest: basic demographic information, clinical and motor assessment, TMS-EMG parameters of cortical excitability, cognitive and neuropsychiatric evaluations, electrophysiology and neuroimaging examination, and serum biomarker testing. Each measure will be collected before and after treatment for both the real and sham groups and during the two phases of the study. Primary, secondary, and exploratory outcome measures are summarized in the Table 1.

**Table 1:** Primary, secondary, and exploratory outcome measures.

	Measure	Domain measured
**Primary outcomes**		
Clinical improvement	MDS-UPDRS (part II, III, IV)	Severity and progression of PD symptoms
**Secondary outcomes**		
Clinical state	Demographic, medical and clinical records	Age, education, handedness during activities of daily living, medical history, treatments, disease duration, comorbid diseases or disorders, etc.
	LEDD	LEDD calculated as a sum of each parkinsonian medication
	PFS-16	Fatigue and impact on daily activities
	PDQ-39	Frequency for difficulties because of PD in last month
Cognitive state	MMP	Screening of cognitive function
	FAB	Frontal Lobe function related activities
	BVRT	Visuospatial memory
Neuropsychiatric state	BDI-II	Depression
	HAM-A	Anxiety
	SAS	Apathy
	SEND-PD	Psychosis, apathy, impulse control behaviors
	PPQ	Psychosis (hallucinations/illusions, delusions, spatiotemporal disorientation)
	FrSBe	Behavior related to frontal system dysfunction
	QUIP	Impulse control disorder behaviors
	CNS-LS	Emotional lability
Electromyography	Cortical excitability TMS-EMG measures	Motor thresholds: rMT, aMT, 1 mV threshold (20 t); cSP
**Exploratory outcomes**		
Electrophysiology	Resting-state EEG activity	EEG PSD and connectivity measures, synchronization measures from graph theory metrics.
Neuroimaging	Structural MRI - T1-weitghted (T13D)	Global and regional GM volume changes and CT computation changes
	Resting-state fMRI	Seed-to-voxel and seed-to-seed functional connectivity measures
Serum protein quantification	Single-molecule assay (Simoa; SR-X™ biomarker detection system)	NfL levels
		GFAP levels
		BDNF levels

aMT, active motor threshold; BDI-II, beck depression inventory; BDNF, brain-derived neurotrophic factor; BVRT, Benton visual retention test; CNS-LS, center for neurology study-lability scale; cSP, contralateral silent period; CT, cortical thickness; EEG, encephalography; EMG, electromyography; FAB, frontal assessment battery; fMRI, functional magnetic resonance imaging; FrSBe, frontal systems behavior scale; GFAP, glial fibrillary acidic protein; GM, gray matter; HAM-A, Hamilton anxiety rating scale; LEDD, levodopa equivalent daily dose; MDS-UPDRS, the movement disorder society-sponsored revision of the unified Parkinson’s disease rating scale; MMP, mini mental Parkinson; MRI, magnetic resonance imaging; NfL, neurofilament light chain; PD, Parkinson’s disease; PDQ-39, Parkinson’s disease questionnaire; PFS-16, Parkinson fatigue scale; PPQ, Parkinson’s psychosis questionnaire; PSD, power spectral density; QUIP, questionnaire for impulsive-compulsive disorders in Parkinson’s disease; rMT, resting motor threshold; SAS, Starkstein apathy scale; SEND-PD, scale for the evaluation of neuropsychiatric disorders in Parkinson’s disease; TMS, transcranial magnetic stimulation.

#### Demographic information and clinical records

- Medical record: medical history, treatments, disease duration, comorbid diseases or disorders and/or any related information to the medical dimension.
- Edinburgh handedness inventory (EHI): screening test to ascertain the subjects’ handedness during activities of daily living (ADL; Oldfield, 1971).
- Modified Hoehn and Yahr Scale (H&Y): a scale used to describe the progression of the disease by descriptive staging. The stages are as follows: (0) no signs of disease, (1) unilateral involvement only, (1.5) unilateral and axial involvement, (2) bilateral involvement without impairment of balance, (2.5) mild bilateral disease with recovery on pull test, (3) bilateral disease with mild to moderate disability and impaired postural reflexes but physically independent, (4) severely disabling disease but still able to walk or stand unassisted, and (5) confinement to bed or wheelchair unless aided (Hoehn and Yahr, 1967; Goetz et al., 2004).

#### Clinical variables

- The movement disorder society - unified Parkinson’s disease rating scale (MDS-UPDRS; part II, III & IV) scale: a tool for rating the severity and progression of typical PD symptoms. Only the motor experiences of daily living (II), motor examination (III), and motor complications (IV) subscales will be administered (Goetz et al., 2008; Postuma et al., 2015).
- Levodopa equivalent daily dose (LEDD): Calculations of the LEDD will be based on a previously reported conversion formula that has been successfully used to determine daily dose equivalents (Tomlinson et al., 2010; Jost et al., 2023).
- Parkinson fatigue scale (PFS-16): a tool used for the evaluation of the physical aspects of fatigue due to parkinsonism and its impact on the patient’s daily activities. Emotional and cognitive dimensions are not included, as they manifest independently of PD. Two scoring methods are proposed, although we used method 1 (Brown et al., 2005).
- Parkinson’s disease questionnaire (PDQ-39): the most widely used rating scale for Parkinson’s disease that addresses the frequency with which patients have experienced any difficulties in the last month because of having PD. It contains 39 items organized into 8 dimensions: mobility, activities of daily living, emotional well-being, social stigma, social support, cognition, communication, and physical discomfort (Peto et al., 1995).

#### Cognitive assessment

- Mini mental Parkinson (MMP): a brief questionnaire derived from the mini mental state examination and used for the assessment of cognitive function in PD patients. The MMP is composed of seven different sections: temporal and spatial orientation, visual registration, attentional/mental control, two-set verbal fluency, visual recall, shifting, and concept processing (Mahieux et al., 1995).
- Frontal assessment battery (FAB): a short battery consisting of 6 subtests related to different frontal lobe functions. These functions are as follows: (1) conceptualization and abstract reasoning, (2) mental flexibility, (3) motor programming and executive control of action, (4) sensitivity to interference, (5) inhibitory control, and (6) environmental autonomy (Dubois et al., 2000).
- Benton visual retention test (BVRT): a test used for the evaluation of visuospatial memory (Benton, 1978). In our protocol, we plan to use the multiple choice format of administration, which consists of stimulus presentation followed by concealment for immediate recognition of one item out of four answer options (specifically, the M form; Le Carret et al., 2003; Amieva et al., 2006).

#### Neuropsychiatric assessment

- Beck depression inventory (BDI-II): a self-report questionnaire developed for the evaluation of depressive symptomatology severity. Affective, cognitive, somatic, and vegetative symptomatology are assessed by the 21 items that comprise this inventory. Scores are used to classify individuals as having minimal, mild, moderate, or severe depression (Beck et al., 1996).
- Hamilton anxiety rating scale (HAM-A): a 14-item clinician-administered rating scale widely used in clinical and research studies to measure the severity of psychic (mental agitation and psychological distress) and somatic anxiety (physical complaints related to anxiety; Hamilton, 1959).
- Starkstein apathy scale (SAS): a 14-item scale used to screen for and determine the severity of apathetic symptoms. These symptoms are as follows: diminished motivation; other aspects related to the behavioral, cognitive, and emotional spheres of apathy; and insight (Starkstein et al., 1992).
- Scale for the evaluation of neuropsychiatric disorders in Parkinson’s disease (SEND-PD): a 12-item scale divided into three subscales: psychotic symptoms, mood/apathy, and impulse control behaviors. Items are rated from 0 (not present) to 4 (very severe; Martinez-Martin et al., 2012).
- Parkinson’s Psychosis Questionnaire (PPQ): a screening tool used to measure the frequency and severity of early signs and psychotic symptoms of PD. These include sleep disturbances, hallucinations/illusions, delusions, and spatiotemporal disorientation (Brandstaedter et al., 2005).
- Frontal systems behavior scale (FrSBe): a self-report scale designed to assess the changes in behavior that may occur in relation to frontal system dysfunction. The FrSBe is composed of 3 subscales: apathy, disinhibition, and executive dysfunction (Grace and Malloy, 2001).
- Questionnaire for impulsive-compulsive disorders in Parkinson’s disease (QUIP): a screening questionnaire developed for the assessment of ICDs and related behaviors. The questionnaire is divided into 3 sections. Section 1 assesses four ICDs (gambling and sexual, buying and eating behaviors), Section 2 assesses related compulsive behaviors (punding, hobbyism and aimless wandering), and Section 3 measures compulsive medication usage (Weintraub et al., 2012).
- Center for neurologic study-lability scale (CNS-LS): a screening instrument composed of seven items that are scored by the patient according to the perceived frequency of pseudobulbar affect (PBA) episodes during the last week (Moore et al., 1997).

#### Electrophysiology

- Electroencephalography (EEG) data: resting-state oscillatory EEG activity will be collected using a high-density 128*-*channel EEG system and recorded during a resting-state session of approximately 10 min that includes both eyes-closed and eyes-open conditions. Participants will be instructed to focus on a fixation cross on the center of a desk in front of individuals during the eyes-open condition.
- Electromyography (EMG) data: several resting-state and active TMS-EMG measures will be calculated for both hemispheres after a single-pulse stimulation is applied over the M1 and collected from the contralateral FDI muscle (see *EMG data analysis* for a detailed description of the analysis that will be performed). The subjects will be instructed to remain relaxed throughout the experiments with the aid of visual feedback from the EMG monitor.

#### Neuroimaging

For all participants, brain MRI data will be collected using a 1.5 T scanner (Siemens Symphony, Erlangen, Germany). The following sequences will be used for data acquisition:

- Structural MRI: high-resolution T1-weighted anatomical images will be obtained using a 3D sagittal MPRAGE sequence (TR = 2.200 ms, TE = 3 ms, flip angle = 8°, matrix = 384 × 512 × 176, voxel size = 0.5 × 0.5 × 1 mm).
- Resting-state fMRI: a T2-weighted functional echo-planar imaging (EPI) sequence sensitive to blood oxygen level-dependent (BOLD) signals will be performed with the following parameters during the eyes-open resting state: repetition time/echo time = 3.000 ms/50 ms, 49 ascending slices, matrix = 64 × 64 × 49, voxel size: 4.06 × 4.06 × 4.05. A total of 120 volumes will be acquired with each scan lasting approximately 7 min.

#### Serum biomarkers

Blood samples from PD patients will be collected via venipuncture at each pre- and post-iTBS timepoint of the study by nurse clinicians from the Department of Neurology at Puerta del Mar University Hospital. All blood samples will be collected in clot-activating serum separator tubes, allowed to clot at room temperature for 30 min, and centrifuged for 10 min at 1,500 g to separate serum from the whole blood. The resulting serum will be aliquoted and properly stored at −80°C until analysis. All participants will have their blood drawn to later quantify serum protein levels of neurofilament light chain (NfL), glial fibrillary acidic protein (GFAP), and brain-derived neurotrophic factor (BDNF).

### Safety assessment

Although existing research on the use of iTBS to treat PD patients has not identified any significant side effects, patients will be carefully monitored for the recognized possible concerns, such as epilepsy. Each treatment and follow-up session, therefore, will include a safety assessment. Other potential reactions, such as fatigue, dizziness, and headache, will be recorded to evaluate the safety of iTBS for PD patients. The most frequent side effects of the EEG recordings are expected to be headache and pain or discomfort in the scalp. Pain and hematoma at the blood sampling site, as well as a vasovagal reaction, syncope, or fainting, may be the most frequent side effects after blood collection. Following each real or sham iTBS phase, safety questionnaires will be administered to the patients, and the results will be recorded and analyzed according to severity, seriousness, and causality. Patients’ vitality, physical health, and mental health will also be monitored to assess any potentially major ill effects.

### Data management

Prior to participation, patients will have to agree to the conditions described in the informed consent form. Agreement to participate can be revoked any time during the course of the experiment. All personal information that might violate the privacy of patients will be codified and entered into a secure database. Copies of paper data forms will be kept behind locks in the research lab. All personal, clinical, and functional data will be properly stored into the DIRAYA platform, a system used in the Andalusian Health Service to support electronic medical records, to which only clinicians have access. Furthermore, analysis of resulting neurocognitive and functional data, such as EEG, MRI, and TMS-EMG data, will be stored in a network-attached hospital storage system to keep extra security copies of imaging data. Blood samples of each patient will be stored and transferred (after obtaining the formal written consent of patients) to the facilities of the Biobank of the Andalusian Public Health System in the Puerta del Mar University Hospital.

### Data collection and analysis

Figure 1 provides a visualization of the complete experimental design including the two phases as well as the washout period and crossover process. Recruitment will be managed by three experienced neurologists who, based on patients’ clinical reports and interviews, will determine their suitability for participation in this investigation. Phase I and Phase II will be identical but differ in terms of groups (i.e., patients who received real iTBS will subsequently receive sham iTBS and vice versa). Patients will be randomly assigned to two equal groups (real or sham) in blocks. Data collection and iTBS sessions will not be performed by the same person to ensure blinding. The full protocol and data acquisition will be performed at the Puerta del Mar University Hospital.

**Figure 2:**
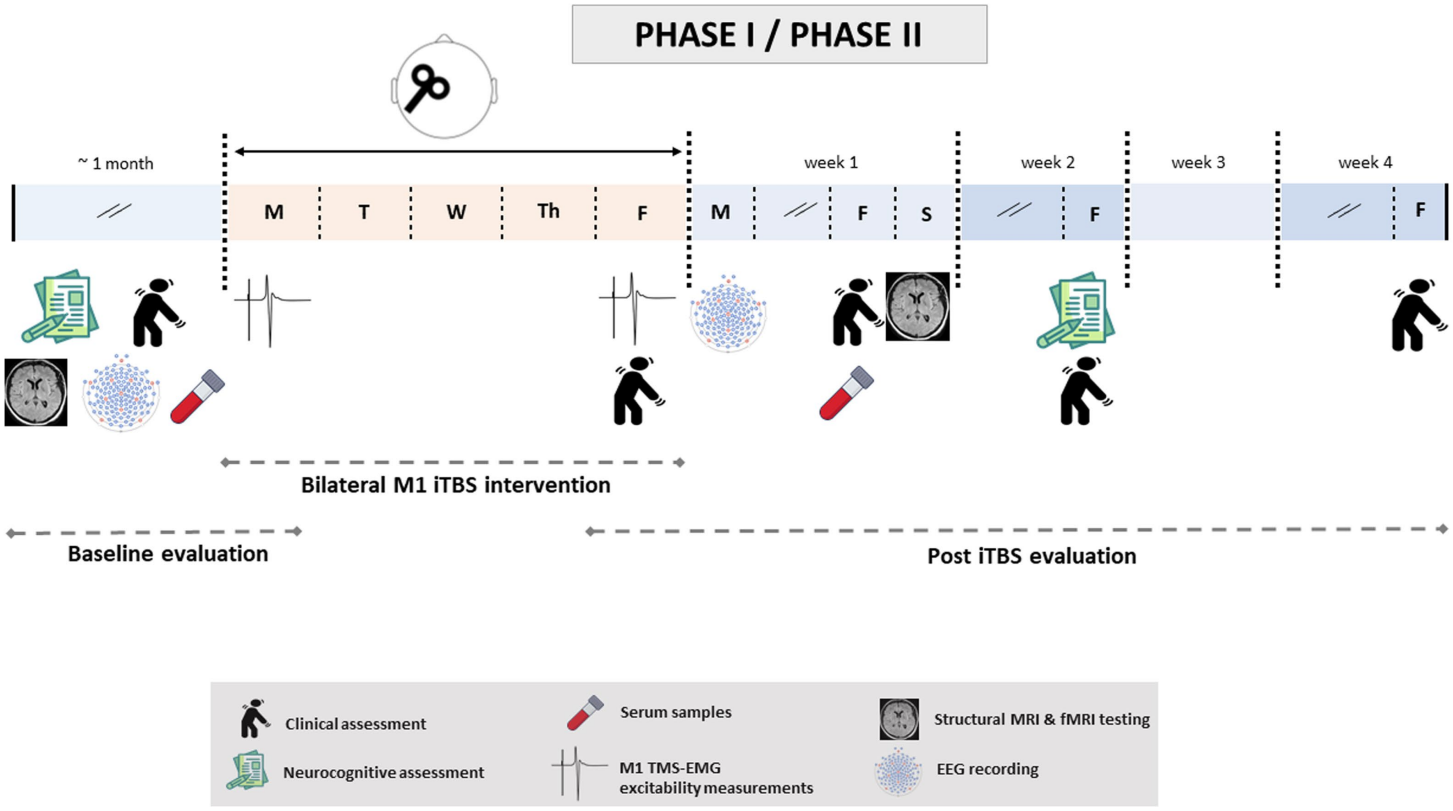
Timeline of study-related events during Phase I/II. After randomization, patients will undergo baseline evaluations. This first examination will include clinical, cognitive and neuropsychiatric evaluations; structural and functional resting-state neuroimaging; EMG; EEG; and blood sample collection. These tests will be performed over a maximum of 1 month and prior to the first iTBS session, with the exception of EMG recording (which will be carried out on the same day, immediately before this first session). Once baseline evaluations are completed, patients will undergo iTBS intervention: one daily (real or sham) iTBS session over the bilateral M1 at 80% of the aMT for five consecutive days. Subsequently, the post-iTBS evaluation stage will start with TMS-EMG data acquisition and clinical examination. The rest of the tests will be performed in the following 4 weeks after the last TMS session (week 1: EEG, clinical evaluation, serology and neuroimaging; week 2: cognitive and neuropsychiatric evaluations, clinical evaluation; week 4: last clinical exploration). The same timeline will be followed for Phases I and II.

Figure 2 provides a summary of the approximate schedule of patient data acquisition in both phases. Once data are collected, we will determine differences in all outcome parameters before and after intervention and between the real and sham stimulation groups.

#### Clinical, cognitive and neuropsychiatric assessments

A neurologist specializing in movement disorders and PD and certified through the MDS-UPDRS Training Program will be responsible for blindly performing all evaluations of the patients’ clinical condition during the different phases of our study protocol. Patients will be assessed at approximately the same time of day to control the effects of medication and, more specifically, to ensure an optimal on-medication state. The neuropsychiatric and cognitive assessments will be performed by two qualified neuropsychologists. The administration of these tests as well as the scoring and correction of data will be performed according to the specific international recommendation for each test and following the Spanish adaptation guidelines. The resulting clinical, neurocognitive, and neuropsychiatric data will be compiled in a database.

#### Electrophysiology data

##### Resting-state EEG activity

Two researchers will be in charge of EEG recordings. High-density EEG data will be collected and recorded using a 128-sponge Ag/AgCl electrode Geodesic Sensor Net (HydroCel GSN, Magstim-EGI, Oregon, United States). EEG signals will be sampled at 1 kHz and recorded using Net Station v. 5.4 software (Magstim/EGI Inc.) with an EGI Net Amps 400 high impedance amplifier. Electrode impedances will be monitored online, and a notch filter will be applied at 50 Hz only for data visualization to remove power line noise. EEG data will be processed offline using the Fieldtrip toolbox (Oostenveld et al., 2011) and custom MATLAB routines (MathWorks, Natick, MA). Briefly, continuous EEG data will be bandpass filtered (0.5–98 Hz) and then segmented into 2-s epochs. EEG signals will be visually inspected, and epochs containing high amplitude artifacts related to participant movements or showing excessive blinks will be rejected. Furthermore, independent component analysis (ICA) will also be conducted to identify and eliminate the components of the EEG signal corresponding to artifacts due to eye movements (saccades and/or blinks) or electrocardiogram activity. The final sample will consist of at least 4 min of artifact-free data. Subsequently, power spectral density (PSD) values will be obtained after fast Fourier transformation (FFT) and averaged to compute an averaged FFT power spectrum for each electrode. PSD values for the delta, alpha, beta, theta, and gamma bands will be analyzed and computed for each treatment phase and stimulation condition.

Individual alpha frequency (IAF) values will be estimated from the mean spectrum over posterior scalp sites by means of peak detection between 7 and 14 Hz. The mean spectral amplitude within the frequency range of the IAF ± 2 Hz (Klimesch, 1999) will be calculated and log-transformed. Thus, PSD values for the following bands will be computed and compared according to each treatment phase and stimulation condition: delta (1–4 Hz), theta (4– to the minimum between 7 Hz and IAF–2 Hz), low alpha (IAF–2 Hz to IAF), high alpha (IAF to IAF +2 Hz), low beta (13–20 Hz), high beta (20–35 Hz), low gamma (35–60 Hz) and high gamma (60–90 Hz; Klimesch, 1999; Babiloni et al., 2020).

Spectral estimates of the relative power of each frequency band during baseline and post-iTBS at any electrode will then be obtained for each participant by subtracting the log-transformed band power during the eyes-closed and eyes-open baseline conditions from the log-transformed band power after treatment, according to the following formula:

Relative frequency band power (*i*) = log (power_i, posttreatment_) – log (power_i, baseline_).

Finally, synchronization measures, such as imaginary coherence (Avena-Koenigsberger et al., 2018) with sensor and source EEG space, will be calculated by obtaining different graph theory metrics to determine the association between graph theory metrics of EEG resting-state brain connectivity and real or sham iTBS treatments. Graph theory measures will be calculated with the igraph package^1^ for the R toolbox (R Core Team, 2018) and Brainstorm software (Tadel et al., 2011; Niso et al., 2019). Resting-state EEG power and EEG connectivity analysis will also be performed by parametric and nonparametric permutation tests to calculate the pre vs. post and real vs. sham differences.

##### TMS-derived measures of cortical excitability

Two researchers and a neurologist, blinded to group allocation, will be responsible for recording bilateral TMS-EMG data to establish stimulation parameters for the iTBS intervention and determine cortical excitability measures.

Surface EMG data will be recorded after a contralateral single-pulse TMS of the FDI muscle with disposable pregelled Ag/AgCl electrodes (EL503, BIOPAC Systems, Inc., California, United States) in a tendon-belly arrangement. Each participant’s skin will be cleaned with a 70% isopropyl alcohol swab to reduce impendences (<5 kΩ) related to skin conductance.

EMG data will be collected using an EMG amplifier and recorded using a BrainAmp ExG amplifier (Brain Products GmbH, Gilching, Germany). For visual display, we will use some filters (bandpass filter: 10–1,000 Hz, sampling rate: 1 kHz) to remove low-frequency artifacts that occur during stimulation; however, raw data will be recorded. All these measures will be visualized and recorded using BrainVision Recorder 2.1 software (Brain Products GmbH, Gilching, Germany).

For EMG analysis, data will be filtered (bandpass filter of 20 Hz to 450 Hz) and digitized at a rate of 1 Hz. The notch filter will be set at 50 Hz to remove power line interference. Different parameters will be obtained from the electromyography session using Analyzer 2.1.2 software (BrainVision Analyzer, Brain Products GmbH, Gilching, Germany) and custom routines and MATLAB toolboxes.^2^ Subsequently, data will be segmented by markers to delimitate trials. In our case, each single TMS pulse will be a marker. Subsequently, baseline correction and artifact rejection will be performed. Normalized EMG data will be used for subsequent statistical analysis.

The following TMS-EMG parameters of cortical excitability will be obtained after stimulation of the FDI muscle hotspot of the mapping grid:

- The resting motor threshold (rMT) of the relaxed FDI muscle will be determined by the modified relative frequency method, under which the rMT is defined as the minimum stimulation intensity required to produce a peak-to-peak MEP amplitude ≥50 μV in at least five out of ten consecutive trials when single pulses are applied to the target area and while the patient is in a resting state (Rossini et al., 1994).
- The active motor threshold (aMT), which is defined as the minimum stimulation intensity needed to produce a peak-to-peak MEP amplitude of 200 μV from the contralateral FDI muscle in 50% of the trials when ten consecutive single pulses are applied with the TMS while the patient is actively contracting the target muscle (20% of maximal voluntary contraction, as determined by visual feedback; Rothwell et al., 1999; Huang and Rothwell, 2004). The 80% stimulation intensity threshold for the aMT will be used to determine parameters for iTBS treatment (Huang et al., 2005).
- To ameliorate the impact of the variability in cortical excitability parameters such as MEP amplitude and MEP onset (Rossini et al., 1994), twenty single pulses (“20 t”) with an interpulse interval of 4–5 s will be given at the minimum intensity able to evoke 1 mV peak-to-peak MEPs in five out of ten consecutive trials from the relaxed contralateral FDI muscle (Bagnato et al., 2006; Suppa et al., 2014)
- The cortical silent period (cSP) will be determined by recording 10 single pulses separated by 10 interpulse seconds and delivered at an intensity of 140% of the rMT while the patient is maintaining a constant voluntary contraction of the contralateral FDI muscle (at 20% of maximal voluntary contraction, as determined by visual feedback; Farzan, 2014).

Aside from these motor threshold values, cortical excitability analysis will include the integration and examination of multiple parameters obtained from the 20 t and cSP recordings. For the analysis of the 20 t trials, a visual inspection will be performed to reject MEPs considered to be outliers. Considering the large fluctuation in amplitude response when recording a cascade of MEPs with a threshold intensity (Rossini et al., 1994), motor evoked potentials will be discarded when their peak-to-peak amplitude is <200 μV. From the 20 t recording, a total of three parameters will be obtained: MEP onset, peak-to-peak amplitude, and MEP area.

- MEP onset (ms): defined as the time between the application of a single TMS pulse and the change of the constant EMG baseline with increasing amplitude. For the determination of the 20 t MEP onset, the lowest latency from the most representative group of onsets of all twenty trials will be selected (Groppa et al., 2012).
- Peak-to-peak amplitude (mV): defined as the average distance between the maximum positive peak and the maximum negative peak in the waveform of the total plotted MEPs.
- MEP area (μV/ms): defined as the area under the rectified MEP curve counting from onset to the offset previously established for the non-rectified averaged MEP. Thus, MEP Area onset will be defined as the beginning of the MEP, and offset will be defined as the return of MEP activity to baseline.

For the cSP recording, a total of five measures will be calculated: cSP duration, MEP peak-to-peak amplitude, MEP area, cSP ratios (duration/amplitude and duration/area; Orth and Rothwell, 2004). A visually guided manual method will be used for inspection. Mean cSP values will be plotted based on trial-by-trial measurements.

- Absolute cSP duration (ms): average duration from the MEP offset to the resumption of sustained EMG activity. The resumption of EMG activity determines the end of cSP (Orth and Rothwell, 2004; Oliviero et al., 2005, 2006; Groppa et al., 2012; Farzan, 2014).
- MEP amplitude: mean peak-to-peak amplitude of the MEPs before the cSP.
- MEP area: area under the rectified MEP curve (Orth and Rothwell, 2004).
- cSP ratio I: calculated as cSP duration/MEP amplitude (Orth and Rothwell, 2004; Oliviero et al., 2006).
- cSP ratio II: calculated as cSP duration/MEP area (Orth and Rothwell, 2004).

Finally, rMT, aMT, 20 t and cSP values will be entered in a database and subsequently analyzed in SPSS.

#### Structural and resting-state functional MRI

Structural and functional MRI analyses will be conducted by two experienced imaging scientists with solid knowledge of advanced volumetric and surface structural procedures and resting-state functional connectivity (rs-FC) analysis. After the MRI data are collected, images will be converted into NIfTI format. Subsequently, a visual inspection will be performed for the detection of artifacts, and they will be manually aligned along the anterior and posterior commissure (AC & PC) with Statistical Parametrical Mapping (SPM12).^3^ Four different analyses will be performed to examine cerebral structural and functional integrity.

Gray matter (GM) volume quantification will be obtained following voxel-based morphometry (VBM) methods using the Computation Anatomy Toolbox (CAT-12, version 12.7) with the current version of SPM12. For this purpose, 3D T1-weighted images will first be visually inspected for artifacts. Subsequently, preprocessing will be performed following standard steps suggested in the CAT-12 manual: (1) bias-field correction; (2) segmentation into GM, white matter (WM), and cerebrospinal fluid (CSF); (3) registration to a standard template using the DARTEL algorithm; (4) normalization of GM images to the Montreal Neurological Institute (MNI) template; and (5) modulation and smoothing of normalized data using an 8 mm FWHM Gaussian kernel. GM volumes of cortical regions of interest will be extracted and corrected for total intracranial volume using the Neuromorphometrics atlas.^4^

Cortical thickness (CT) computation will be obtained using the CAT-12 toolbox, which follows an automated method of tissue segmentation based on the estimation of distances between WM and GM voxels using a projection-based thickness method, including topology correction and spherical mapping. Additionally, individual cortical surface maps will be smoothed with a 15 mm Gaussian kernel to allow intersubject comparisons. Regional CT values will be extracted using the Desikan-Killiany atlas (Desikan et al., 2006).

Independent component analysis (ICA) of rs-FC data. Resting-state fMRI images will be preprocessed using the Data Processing & Analysis for Brain Imaging tool (DPARSF V4.3).^5^ Preprocessing will include removal of the 10 first functional volumes, slice-timing correction, realignment of the first volume, coregistration, segmentation, spatial normalization to MNI space and spatial smoothing (with a 4-mm FWHM Gaussian kernel). Furthermore, ICA will include the estimation and extraction of brain networks of interest following the infomax algorithm, performed with the Group ICA toolbox of fMRI Toolbox (GIFT) software.

Seed-based rs-FC. Resting-state fMRI data will be preprocessed using the Data Processing & Analysis for Brain Imaging tool (DPARSF V4.3; footnote 5). Preprocessing will include removal of the 10 first functional volumes, slice-timing correction, realignment to the first volume, head-motion correction, coregistration, nuisance covariate regression, spatial normalization to MNI space, spatial smoothing (with a 4-mm FWHM Gaussian kernel), and temporal filtering (0.01 Hz – 0.1 Hz). Seed-to-voxel and seed-to-seed FC values will be computed. The brain regions with significant structural differences between the real and sham iTBS conditions will subsequently be used as seeds to trace the brain regions associated with abnormal FC.

#### Quantification of serum brain biomarkers

Serum quantification of protein biomarkers will be determined by a biologist and a laboratory support technician with training on structured protocols that will include extensive supervised practice and performance-based certification. All measurements will be assessed via ultrasensitive single molecule array (Simoa™) technology through the SR-X Instrument (Quanterix Corporation, MA, United States). Serum NfL and GFAP levels will be measured using the Neurology 2-Plex B assay, and BDNF levels will be measured using the BDNF Discovery kit (Quanterix, Corporation MA, United States). All data will be analyzed according to the manufacturer’s instructions and recommendations for blood biomarkers.

### Statistical analysis

Before any statistical analyses are performed, the normality of the data distributions of outcome variables and parameters will be assessed using the Shapiro–Wilk test to determine whether parametric or nonparametric statistical tests should be used for the different measures in this study. Subjects will act as their own controls, and the data will be analyzed using both parametric (independent-samples *t*-test) and nonparametric (Mann–Whitney U test) methods, as appropriate. To evaluate the carryover effects, a preliminary test will be performed to test the assumption that the washout phase lasted long enough to completely eliminate a carryover impact. To do this, an independent-samples test will be used to compare the sum of the values measured in the two periods for each subject across the two sequence groups.

In the first step, exploratory analysis of correlations between the clinical response variables and all outcome measures will be conducted. Primarily, correlation analyses will be applied to examine correlations of TMS-EMG parameters with motor, clinical, and neurocognitive variables as well as serum biomarkers and brain structural and functional variables. In a second step, significant correlations across timepoints (i.e., use of baseline variables to predict the clinical response at posttreatment) will be analyzed using stepwise multiple regression models to identify the potential factors predicting the clinical response after iTBS.

For structural MRI and functional (EEG, fMRI) data, individual analysis will first involve the statistical pipeline of each software or toolbox (e.g., SPM12, Fieldtrip, Brainstorm, etc.). Next, advanced statistical analysis will be performed by incorporating clinical, cognitive, neuropsychiatric, serum biomarker, electrophysiological, and neuroimaging information using SPSS 23.0 (IBM, Chicago, IL, United States), R, and custom routines written in MATLAB (MathWorks, Natick, United States). Issues affecting the risk of bias related to missing data in our crossover study will be considered. Initially, all patients with missing outcome data will be removed from the analysis of each data subset. Other strategies for managing missing data, such as the last observation carried forward, will also be used to impute values. The threshold for statistical significance will initially be set at *p* < 0.05. All statistical tests will be two-tailed and adjusted for multiple comparisons.

### Adherence and dropout

At the start of each stimulation session, the patient will confirm their willingness to participate. The patient will indicate their interest in taking part in our study before each stimulation session, and they will be free to leave at any moment. During the patient’s participation in the study, the research clinician will ensure that the patient is still eligible to participate. An individual will be removed from the study upon losing eligibility or reporting any major negative side effects.

Thus, patients will be asked to drop out of the investigation if any adverse effect occurs after stimulation or if they fail to cooperate to the extent that the normal protocol of the study cannot be carried out. Patients will be free to drop out at any point if they so desire. To improve adherence to the study procedures, telephone follow-up will be carried out during the washout phase so that any adverse consequences can be properly managed. Moreover, if any brain abnormalities are observed during the MRI (e.g., white matter hyperintensities, cerebral vascular lesions, or evidence of space-occupying lesions) or EEG assessments (e.g., epileptiform activity, ictal patterns) or after blood testing (e.g., exaggerated levels of sNfL), the patient will be referred to the proper medical services for further analysis and consideration, which will inform the decision of whether the patient may continue in the study.

Although rescue treatment will not initially be required, nonresponders will have the option to receive another 5 sessions of real iTMS bilaterally over M1 at the end of the study as an add-on rescue treatment. Patients and their relatives will be instructed to maintain their normal daily routines and not to alter their physical exercise patterns, diet, sleep schedules, or any other nonpharmacological treatment throughout the study, as well as to report any change.

Dopaminergic medication for all patients will be stabilized for at least 2 months prior to the study. Patients will need to maintain their dosage of dopaminergic medication until the completion of Phase II. In addition, assessment of nondopaminergic medications will be conducted to determine their influence on the tests performed.

## Discussion

PD is a progressive disorder caused by dopamine depletion, which contributes to the manifestation of characteristic related clinical symptomatology. Improving motor and non-motor functional outcomes is therefore a priority in the management of PD patients. Although a wide range of treatments is available, PD symptoms may progress, leading to severe impairment. The pharmacotherapy of PD relies on dopamine level restoration, and although good management of symptomatology is commonly achieved, this balance is maintained for only a short period of time, as complications related to medication may occur, thus impacting tolerability and treatment efficacy (Jankovic, 2002; Tambasco et al., 2018). Numerous therapeutic alternatives have been developed to ameliorate the symptom impact and disability associated with the disease and its first-line pharmacology options (Mishima et al., 2021; Serva et al., 2022). In fact, different rTMS modalities are considered a potential therapy for PD, given the safety and lack of side effects after application (Wagle Shukla et al., 2016). Nevertheless, although a variety of rTMS protocols have been demonstrated to be effective, many of them have limited benefits in terms of time and effects (Chou et al., 2015; Zanjani et al., 2015); thus, an increasing number of studies has examined the therapeutic impact of TBS, which has a shorter stimulation duration and more intense stimulation sequence, on motor and nonmotor symptoms in PD patients (Cheng et al., 2022). However, inconsistent conclusions have been reported because only a few TBS studies have been performed; hence, the magnitude and persistence of the TBS effect remain debatable.

Most previous studies have supported the clinical efficacy of cTBS over the SMA for improving motor function in individuals with PD in the OFF medication state, but the clinical effect and ultimate clinical importance of iTBS over the M1 remain unclear and controversial. Thus, while a single session of cTBS over the M1 did not have any motor or clinical effects in PD patients in the OFF medication state (Eggers et al., 2010), a study using a single session of iTBS over the M1 was promising for alleviating complications in PD patients in the ON medication state (Degardin et al., 2012). However, some disagreements regarding the application of excitatory or inhibitory TBS protocols and the recommended brain targets during the ON medication state still need to be clarified (Cheng et al., 2022).

There is broad consensus that TBS protocols, like other types of rTMS, may promote cortical plasticity that treats disease, improves health, and modifies brain activity. Moreover, evidence indicates that the after-effects of TBS protocols are due to underlying mechanisms of synaptic plasticity. Accordingly, LTP and LTD are thought to be the processes underlying the stimulation and suppression of MEPs by iTBS and cTBS protocols, respectively. In this regard, the restoration of post-TBS MEP amplitudes to their baseline levels is a neurophysiologic indicator of the clinical effectiveness and efficacy of the mechanisms underlying cortical plasticity (Oberman, 2010; Pascual-Leone et al., 2011).

However, it is important to note that LTP and LTD pertain to extremely specific neurophysiologic events at the level of individual synaptic connections, and the alterations induced by TBS are not always limited to specific individual synaptic terminals. It is more probable that many of the positive effects of TBS are the result of the secondary activation of glial cells, although there is limited information regarding the response of glial cells to these neuromodulation techniques (Cullen and Young, 2016), particularly in relation to iTBS-induced plasticity effects (Cacace et al., 2017). Moreover, knowledge about how intracortical structures, cortico-subcortical functional connectivity, and brain volume changes are involved in the modulatory effects of rTMS and TBS is still limited (Di Lazzaro and Rothwell, 2014; Peters et al., 2020; Jannati et al., 2023; Kirkovski et al., 2023).

Although the postulated mechanism of iTBS (i.e., stimulating neuronal and glial activity) has been demonstrated to prompt LTP-like plasticity *in vivo* (Funamizu et al., 2005; Cullen and Young, 2016; Cacace et al., 2017), human studies targeting microglia and astrocyte reactivity in areas affected by neurological or psychiatric diseases are scarce. Thus, it has been hypothesized that the benefits of iTBS for reducing motor and nonmotor symptomatology in patients with PD, as well as many other brain-related diseases, might be due to effects on microglia and astrocytes, which play a protective role in neurodegenerative and neuroinflammatory diseases (Stanojevic et al., 2022). In line with the quantification of blood biomarkers of neurodegeneration, some recent studies have reported that excitatory frontal and parietal rTMS, in addition to improving cognition, is not associated with neuroaxonal damage or neurodegeneration in healthy populations according to plasma NfL concentrations (Redondo-Camós et al., 2022); thus, the effects of iTBS on reducing neurodegenerative pathology in animal models and humans still need to be tested. Furthermore, evidence from animal models and neurological patients suggests that TBS and rTMS protocols may induce changes in the regulation of BDNF expression (Zhang et al., 2007; Stevanovic et al., 2019) or changes in BDNF protein levels (Müller, 2000) and promote neuroprotection and neuroplasticity (Feng et al., 2012; Castillo-Padilla and Funke, 2016; Zuo et al., 2020). However, the role of BDNF in the effects of TBS on PD patients is still debated, as it may have a variable response to iTBS compared to cTBS, likely because the PD brain might be less susceptible to iTBS-induced motor plasticity and potentiation (Cheeran et al., 2008). Further investigation of BDNF down/upregulation is essential to better understand the scope and capacity of iTBS-induced plasticity.

While it has been demonstrated that a few sessions of rTMS or TBS can increase GM volume at the stimulation site as well as in more distal brain regions (May et al., 2007), the potential role of cerebral CT as a possible biomarker of iTBS treatment response in PD patients still needs to be assessed. These GM volume changes could demonstrate the substantial neuroprotective effects of rTMS or TBS, likely dependent on a wide range of micro- and macroscopic neural mechanisms (Ji et al., 2021; Jung and Lambon Ralph, 2021). In light of this, metabolic neuroimaging studies have provided additional evidence of neuroplastic changes after rTMS or TBS, as M1 rTMS was shown to influence the resting-state activity of the motor system after the stimulation had ended as well as in brain regions connected to the stimulation site (Lee et al., 2003; Rounis et al., 2005), demonstrating an acute reorganization of activity to other areas. Moreover, studies in PD patients have reported changes in functional connectivity between the M1 and basal ganglia or between the prefrontal areas and the SMA after low-frequency (Wang et al., 2023) or high-frequency rTMS (González-García et al., 2011), respectively. Despite the evidence demonstrating altered brain functional connectivity in PD, conclusive data regarding the potential link between clinical improvement and changes in the brain’s structural and functional connectivity after iTBS in these patients are lacking.

In conclusion, this study is designed to provide new insights that promise to advance our understanding of the efficacy of M1-iTBS in improving symptomatology in the early and middle stages of PD. We hypothesize that bilateral M1-iTBS will considerably improve motor functioning in PD patients, as measured by MDS-UPDRS parts II, III, and IV, in addition to alleviating nonmotor symptoms. A growing body of evidence from preclinical and human studies allows us to further hypothesize that the striatal endogenous dopamine release triggered by iTBS may rebalance cortical excitability and restore compensatory striatal volume changes, increasing the functional connectivity of striato-cortico-cerebellar networks while also positively impacting neuroglia and neuroplasticity modification.

Thus, by identifying potential electrophysiological, neuroimaging and serum markers underpinning iTBS-induced dopamine-dependent corticostriatal plasticity, our study could pinpoint the ideal biomarkers responsible for short- and long-lasting effects on motor function in PD, offering these patients an optimized adjunct to dopamine replacement therapies.

### Study protocol status

This study is currently ongoing and started recruitment in June 2022. The first thirteen patients have been enrolled, and four of them have already undergone the crossover intervention. The estimated completion date is December 2023.

## Summary and conclusions

This randomized double-blind sham-controlled crossover protocol study will investigate the neural and behavioral effects of iTBS over the bilateral M1 on idiopathic PD. The present combination of iTBS, cortical excitability measures, structural and functional neuroimaging data, and serum biomarkers represents a powerful approach that enables noninvasive, *in vivo* assessment and modulation of brain excitability, connectivity, and neuroprotection and neuroplasticity across motor and nonmotor brain networks in PD patients. We hypothesize that bilaterally stimulation of the M1 with excitatory iTBS will yield beneficial clinical effects that improve MDS-UPDRS-III scores in the ON medication state (i.e., motor improvement in PD patients) by modulating regionally specific changes in BG-cortical volume as well as by functional connectivity among the BG, cerebellum and cortical regions involved in motor processing. Moreover, we hypothesize that these functional and structural brain changes will be dynamically associated with the neuroprotective and neuroplasticity effects of real M1 iTBS reflected by stabilization of NfL and GFAP levels and by elevation of BDNF levels. Thus, these results may provide supportive evidence that the bilateral M1 is an optimal excitatory stimulation target for treatment among stable PD patients.

## Ethics statement

The study and the current protocol have been approved by the Andalusian Biomedical Research Ethics Committee (Ref.: 2169-N-19) in accordance with the Code of Ethics of the World Medical Association (Declaration of Helsinki) for experiments involving humans (World Medical Association, 2013). Furthermore, our study aligns with Spanish legislation on the grounds of biomedical investigation (Law 14/2007, July 03), personal data protection (Law 15/1999, December 13) and bioethics. Every participant will provide voluntary written informed consent before participating in our study. The findings will be distributed through (open-access) peer-reviewed publications, networks of scientists and professionals, the general public, patient associations, and presentations at conferences.

## Author contributions

RR-L: Conceptualization, Investigation, Project administration, Visualization, Writing – original draft, Writing – review & editing. PM-G: Conceptualization, Data curation, Formal analysis, Investigation, Methodology, Software, Supervision, Validation, Writing – original draft, Writing – review & editing. FS-F: Writing – review & editing, Data curation, Formal analysis, Investigation, Supervision, Validation, Visualization. FC-C: Data curation, Formal analysis, Investigation, Validation, Visualization, Writing – review & editing. ES-A: Visualization, Writing – review & editing, Data curation, Investigation, Validation. FS: Data curation, Formal analysis, Investigation, Methodology, Software, Supervision, Validation, Visualization, Writing – review & editing. CM-B: Data curation, Formal analysis, Investigation, Methodology, Software, Visualization, Writing – review & editing. EL-S: Writing – review & editing, Data curation, Formal analysis, Investigation, Methodology, Validation, Visualization. ÁG-M: Visualization, Writing – review & editing, Data curation, Investigation, Methodology. LF: Writing – review & editing, Data curation, Funding acquisition, Investigation, Resources, Validation, Visualization. RG-C: Data curation, Investigation, Methodology, Visualization, Writing – review & editing. FL-S: Data curation, Investigation, Methodology, Software, Writing – review & editing, Visualization. AZ: Data curation, Methodology, Visualization, Writing – review & editing. RG-M: Data curation, Investigation, Methodology, Resources, Validation, Writing – review & editing. JG-Ra: Data curation, Formal analysis, Investigation, Methodology, Software, Writing – review & editing. JP-E: Data curation, Methodology, Resources, Writing – review & editing. GR-E: Investigation, Validation, Visualization, Writing – review & editing. RE-R: Data curation, Investigation, Methodology, Resources, Validation, Writing – review & editing. ÁC-G: Data curation, Formal analysis, Investigation, Methodology, Project administration, Software, Supervision, Validation, Visualization, Writing – review & editing. JG-Ro: Conceptualization, Data curation, Formal analysis, Funding acquisition, Investigation, Methodology, Project administration, Resources, Software, Supervision, Validation, Visualization, Writing – original draft, Writing – review & editing.

## Footnotes

^1^ http://igraph.org

^2^ https://github.com/CunninghamLab/TMSAnalysisToolBox

^3^ fil.ion.ucl.ac.uk/spm/

^4^ neuromorphometrics.com/

^5^ http://rfmri.org/DPARSF
